# Hip-related groin pain, patient characteristics and patient-reported outcomes in patients referred to tertiary care due to longstanding hip and groin pain: a cross-sectional study

**DOI:** 10.1186/s12891-019-2794-7

**Published:** 2019-09-14

**Authors:** Anders Pålsson, Ioannis Kostogiannis, Håkan Lindvall, Eva Ageberg

**Affiliations:** 1 Department of Health Sciences, Lund University, PO Box 157, 22100 Lund, Sweden; 2 Department of Orthopaedics, Clinical Sciences, Lund University, Malmö, Sweden; 3 Department of Translational Medicine, Diagnostic Radiology, Lund University, Malmö, Sweden; 4 Department of Imaging and Functional Medicine, Skåne University Hospital Malmö, Malmö, Sweden

**Keywords:** Hip joint, Groin, Pain, Prevalence, Patient reported outcomes

## Abstract

**Background:**

Due to advances in hip arthroscopy, the number of surgical procedures has increased dramatically. The diagnostic challenge in patients with longstanding hip and groin pain, as well as the increasing number of hip arthroscopies, may lead to a higher number of patients referred to tertiary care for consideration for surgery. Therefore, the aims were: 1) to describe the prevalence of hip-related groin pain in patients referred to tertiary care due to longstanding hip and groin pain; and 2) to compare patient characteristics and patient-reported outcomes for patients categorized as having hip-related groin pain and those with non-hip-related groin pain.

**Methods:**

Eighty-one patients referred to the Department of Orthopedics at Skåne University Hospital for longstanding hip and groin pain were consecutively included and categorized into hip-related groin pain or non-hip-related groin pain using diagnostic criteria based on current best evidence (clinical examination, radiological examination and intra-articular block injection). Patient characteristics (gender (%), age (years), BMI (kg/m^2^)), results from the Hip Sports Activity Scale (HSAS), the SF-36, the Copenhagen Hip and Groin Outcome Score (HAGOS), and pain distribution (pain manikin) were collected. Parametric and non-parametric statistics were used as appropriate for between-group analysis.

**Results:**

Thirty-three (47%) patients, (30% women, 70% men, *p* < 0.01), were categorized as having hip-related groin pain. The hip-related groin pain group had a higher activity level during adolescence (*p* = 0.013), and a higher pre-injury activity level (*p* = 0.034), compared to the non-hip-related groin pain group. No differences (mean difference (95% CI)) between hip-related groin pain and non-hip-related groin pain were observed for age (0 (− 4; 4)), BMI (− 1.75 (− 3.61; 0.12)), any HAGOS subscales (*p* ≥ 0.318), any SF-36 subscales (*p* ≥ 0.142) or pain distribution (*p* ≥ 0.201).

**Conclusions:**

Only half of the patients referred to tertiary care for long-standing hip and groin pain, who were predominantly men with a high activity level, had hip-related groin pain. Self-reported pain localization and distribution did not differ between patients with hip-related groin pain and those with non-hip-related groin pain, and both patient groups had poor perceived general health, and hip-related symptoms and function.

## Background

Hip and groin pain is a common problem among athletes participating in high-impact sports [1–4], and can also affect people participating in low-impact activities/sports [5, 6]. Acute hip and groin pain with a sudden onset often only leads to a few weeks of absence from physical activity [1, 3, 7]. However, in long-standing hip and groin pain (LHGP), the symptoms may have a more or less insidious presentation, limiting the person’s ability to participate in physical activities as well as reducing the person’s quality of life [4, 8].

Diagnostics is a challenge in patients with LHGP due to the probable multi-structural origin of the pain where both intra- and extra-articular lesions may be present and even coexist [9, 10]. A recently published consensus statement clarifies the terminology and definitions for describing symptoms presenting in the hip and groin area [11]. In this consensus statement, the following subgroups were agreed upon: 1) groin pain, including adductor-related, iliopsoas-related, inguinal-related, and pubic-related groin pain; 2) hip-related groin pain; and 3) other causes of groin pain [11].

The most common causes of hip-related groin pain are femoroacetabular impingement syndrome (FAI syndrome), chondral lesions, and labral lesions [12, 13]. Femoroacetabular impingement (FAI) is caused by premature contact between the femoral neck and the acetabular rim. This early contact is caused either by the femoral head having an oval shape rather than round (CAM morphology) or by over-coverage of the femoral head by the acetabulum (pincer morphology). This morphological interaction can cause labral tears and chondral lesions due to the changed biomechanics in the hip joint, and is suggested to be a risk factor for developing hip osteoarthritis (OA) [14–16]. However, the presence of CAM and/or pincer morphology is not sufficient to pose the diagnosis of FAI syndrome. The Warwick agreement suggests that a combination of symptoms, clinical signs and radiological findings should be considered when diagnosing FAI syndrome and other pathology related to hip-related groin pain [17]. To further confirm the diagnosis, image-guided intra-articular block injection can be used after all the other criteria have been met [17, 18].

Treatment of hip-related groin pain involves education, modification of activity level, and exercise-based therapy, with the potential benefit of arthroscopic hip surgery [17, 19]. Due to advances in hip arthroscopy, the number of surgical procedures has increased dramatically over the last decade, with a reported increase of between 18- to 25-fold [20, 21]. The diagnostic challenge in patients with LHGP, as well as the increasing number of hip arthroscopy procedures, may lead to a higher number of patients referred to tertiary care for consideration for surgery. This may lead to increase societal costs due to unnecessary investigations [22]. Of those referred to tertiary care, the prevalence of patients with hip-related groin pain who are potential candidates for hip arthroscopy is unclear.

Therefore, the aims of this cross-sectional study were: 1) to describe the prevalence of hip-related groin pain in patients referred to tertiary care; and 2) to compare patient characteristics and patient-reported outcomes between patients categorized as having hip-related groin pain and those categorized as having non-hip-related groin pain.

## Methods

Our reporting for this exploratory cross-sectional study adheres to the STROBE statement (www.strobe-statement.org).

### Participants

**Fig. 1:**
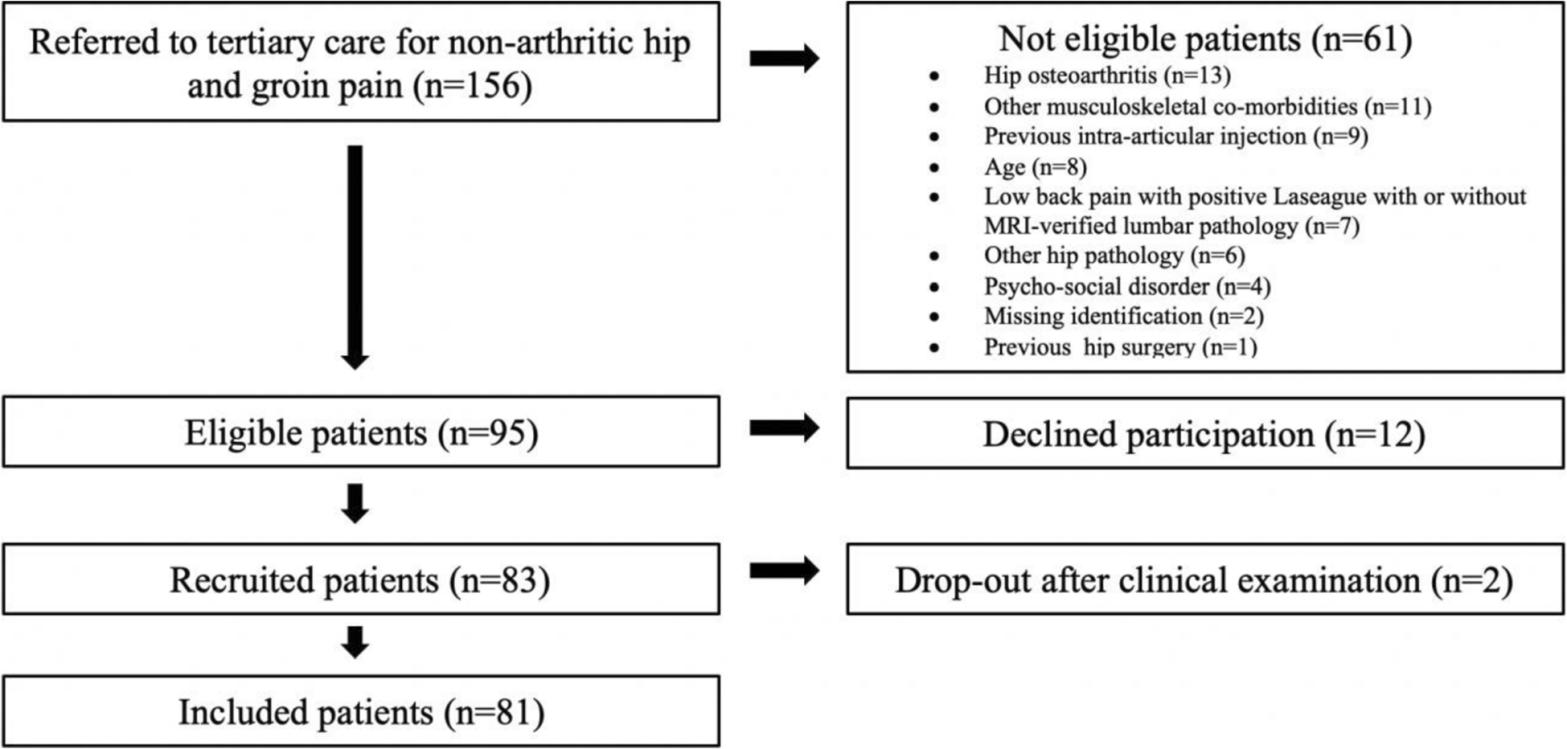
Flow chart of the recruitment process

During October 2014 to January 2017, all patients referred for non-arthritic hip and groin pain (*n* = 156) to the Department of Orthopedics, Skåne University Hospital, Sweden, were consecutively recruited and screened for eligibility according to the inclusion and exclusion criteria as described in Table 1. Ninety-five patients were found eligible, of whom twelve patients declined participation. Eighty-three participants were, consequently, recruited. After the initial clinical examination, two patients declined further participation, and were, thus, excluded. Eighty-one patients were finally included in the study (Fig. 1).

**Table 1:** Inclusion and exclusion criteria for patients referred to tertiary care due to hip and groin pain

Inclusion criteria	
● Unilateral or bilateral hip/groin pain > 3 months	
● Age 18–55 years	
● No previous hip surgery	
Exclusion criteria	
● Other hip pathology (i.e., Perthes disease)	
● Verified moderate or severe osteoarthritis (OA) (Tönnis grade > 1)	
● Patients that had received intra-articular or peri-articular injection with corticosteroids during the last 2 months	
● Palpable hernia	
● Low-back pain with a positive Lasègue test with or without MRI-verified lower back/spine pathology	
● Other musculoskeletal co-morbidities overriding the hip-related symptoms and dysfunction	
● Co-morbidities excluding physical activity and training,	
● Psychosocial disorders	
● Drug abuse	
● Not understanding the language of interest.

### Categorization of hip-related and non-hip-related groin pain

Current best evidence [17] was used to categorize hip-related and non-hip-related groin pain. For patients to be categorized as having hip-related groin pain, the following four criteria had to be met: 1) Passive range of motion (ROM) affected (defined as end-range pain, decreased ROM, or end-range pain + decreased ROM); 2) Pain provocation during at least one hip impingement test; 3) Findings on radiological examination, MRI/MRA or during arthroscopic examination, that are assumed to cause hip-related pain and symptoms [23] (CAM morphology (alpha angle ≥60°), Pincer morphology (lateral center-edge (LCE) angle ≥40°), hip dysplasia (LCE angle ≤20°), acetabular labral tear or chondral lesions); and 4) Responder to diagnostic intra-articular injection (≥50% decrease of pain registered on a visual analog scale (VAS) 0–4 h after injection). If these four criteria were not met, the patient was categorized as having non-hip-related groin pain.

### Clinical assessment

All participants were assessed by a senior orthopedic surgeon specializing in hip arthroscopy. The clinical assessment of the hip included passive ROM and hip impingement tests.

#### Passive hip joint ROM

**Fig. 2:**
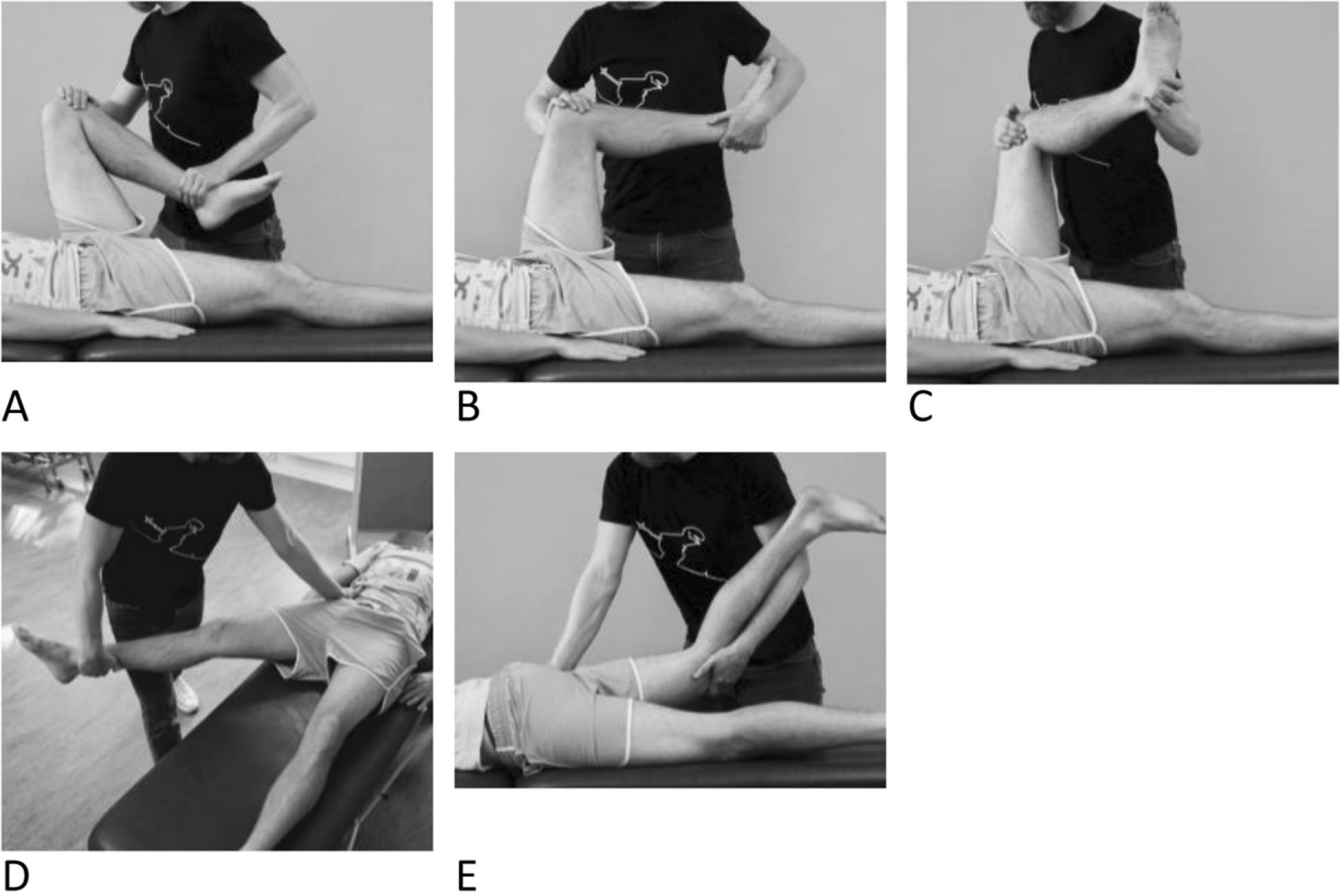
**a-e**. Passive hip ROM in flexion (**a**), medial rotation (**b**), lateral rotation (**c**), abduction (**d**) and extension (**e**)

Passive flexion (Fig. 2a), medial rotation (Fig. 2b), lateral rotation (Fig. 2c) and abduction (Fig. 2d) were examined with the patient in a supine position. Medial and lateral rotation were tested in 90° flexion of the hip and knee joints. Passive extension was examined in a prone position (Fig. 2e). The patient was instructed to stay relaxed during the tests and to report any reproduction of hip/groin pain. We categorized each test in a clinical manner as either 1) negative (defined as full ROM without pain), or 2) positive (defined as end-range pain, decreased ROM, or end-range pain + decreased ROM).

#### Hip impingement tests

**Fig. 3:**
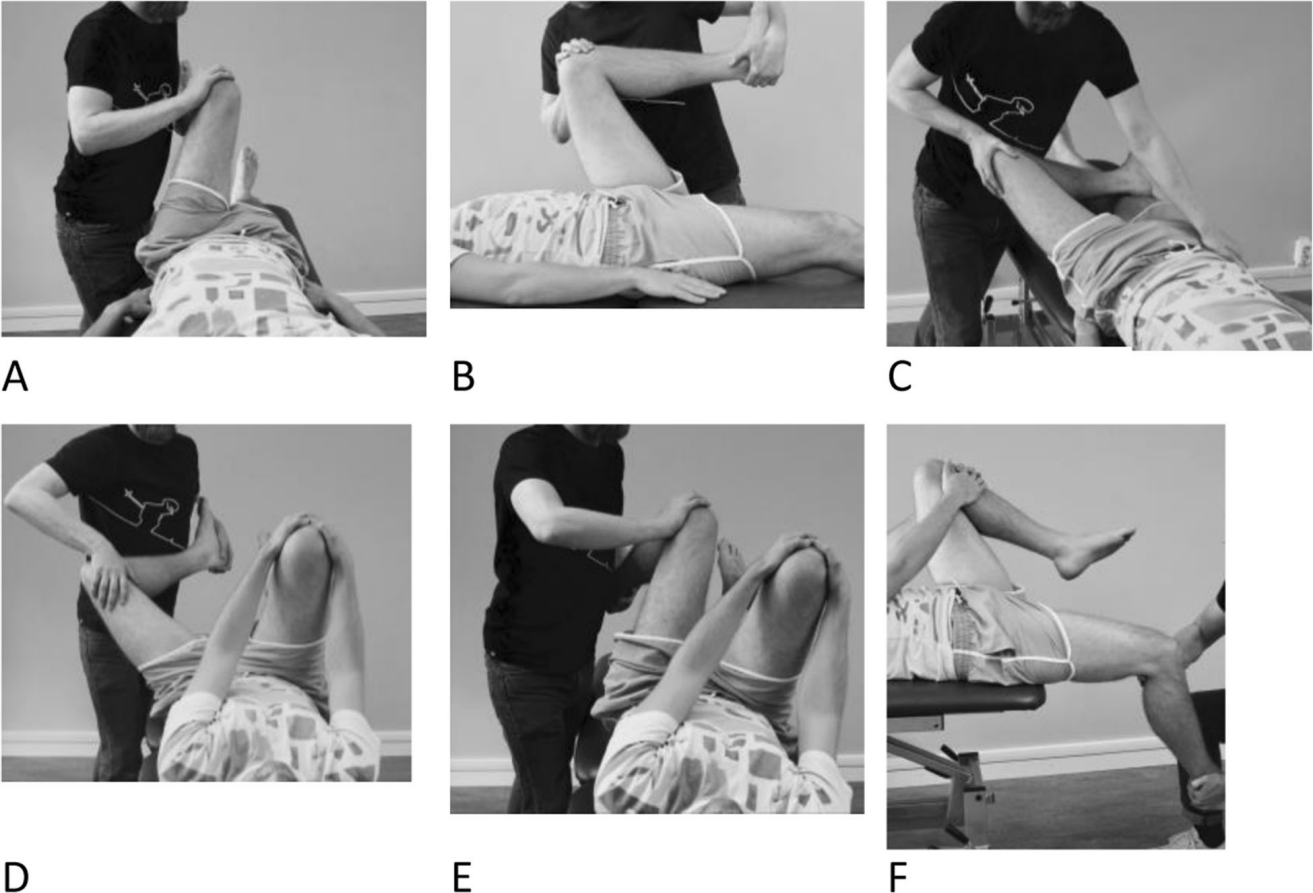
**a-f.** Hip impingement tests: AIMT (**a**) The examiner brings the hip into 90° flexion and then moves the hip into medial rotation and adduction. FADIR (**b**) The examiner brings the hip into maximal flexion, medial rotation and adduction. FABER (**c**) the examined leg is placed with the foot just proximal to the contralateral knee joint and the hip is brought into a combined flexion, abduction and external rotation. The examiner places a hand on the contralateral side of the pelvis to minimize pelvic rotation. DEXRIT (**d**) and DIRIT (**e**) the patient is asked to hold the contralateral hip in more than 90° flexion. The examiner then brings the hip into approximately 90° flexion and moves the hip through a wide arc of extension, abduction and external rotation (DEXRIT) or extension, adduction and internal rotation (DIRIT). PRIMT (**f**) supine position with the patient lying at the edge of the examining table. Both hips are brought into flexion and the patient is instructed to keep the contralateral hip in flexion while the examined hip is brought into extension, abduction and lateral rotation

The following six hip impingement tests were included and performed according to Martin et al. [24]: Anterior Impingement Test (AIMT) (Fig. 3a), Flexion/Adduction/Internal Rotation (FADIR) (Fig. 3b), Flexion/Abduction/External Rotation (FABER) (Fig. 3c), Dynamic External Rotatory Impingement Test (DEXRIT) (Fig. 3d), Dynamic Internal Rotatory Impingement Test (DIRIT) (Fig. 3e) and Posterior Rim Impingement Test (PRIMT) (Fig. 3f). All tests were performed in a supine position. The patient was instructed to report any reproduction of hip/groin pain. The tests were categorized as either 1) negative (no pain), or 2) positive (painful).

### Radiographic data

All radiographs were analyzed by the same radiologist (HL) who was not involved in the care of the patients. The alpha angle and LCE angle were identified and analyzed in accordance with a report by Clohisy et al. [25]. The Lauenstein (frog-leg lateral) projection was used to obtain the alpha angle, whereas the LCE angle was interpreted on the anteroposterior pelvic view.

The alpha angle was calculated by drawing a line from the center of the femoral head to the center of the femoral neck. A second line was drawn from the center of the femoral head to the point where the head loses its spherical appearance antero-laterally. The angle was then calculated between these two lines and values ≥60 degrees were used as the cut-off defining a cam morphology [26]. For five patients no Lauenstein projection was available and the alpha angle was therefore not calculated for these patients.

To calculate the LCE angle, a first line was drawn connecting the inferior part of the acetabular teardrops, and a second line was drawn perpendicular to the first and through the center of the femoral head. Finally, a third line was drawn from the center of the femoral head through the sclerotic part of the superolateral sourcil of the acetabulum. The angle between the second and third line was calculated; an LCE angle ≥40 degrees indicated the presence of a pincer morphology and an LCE angle ≤20° indicated hip dysplasia [26].

In a preliminary analysis of the first 67 patients included in the study, excellent inter-observer agreement was observed between two raters (a medical student and an orthopedic surgeon) using our protocol for measurements of alpha angle (ICC 0.85, 95% CL 0.77–0.91) and LCE angle (ICC 0.84, 95% CI 0.77–0.89) in plain radiographs.

### MRI and arthroscopic examination

Fifty-four patients underwent MRI examination. Records of any labral tear or chondral lesions visual on MRI were extracted from the patient’s medical record. Nineteen patients underwent arthroscopic examination and data on any findings of hip morphology (CAM, Pincer), acetabular labral tear or chondral lesions from the arthroscopic examination were extracted from the patient’s medical record.

### Intra-articular block injection

All injections were performed by the senior orthopedic surgeon under fluoroscopic guidance. The intra-articular position of the needle was confirmed by injection of 1 ml of contrast agent johexol (Omnipaque, 180 mg I/ml) prior to the blockage injection of a mixture containing 2 ml triamcinolon (Lederspan, 20 mg/ml), 4 ml ropivacaine (Narop 10 mg/ml) and 4 ml lidocaine (Xulocain 10 mg/ml). The patients were asked to score pain on a VAS, from 0 (no pain) to 100 (maximal pain) mm prior to injection, and one, two, and four hours later. During this period the patients were instructed to perform activities that would normally provoke pain in order to determine any improvement in symptoms [27]. A decrease in VAS score of 50% or more over a period of 4 h after injection was considered to be a true effect. The patients were categorized as either responder to injection (≥50% decrease of VAS) or non-responder to injection (< 50% decrease of VAS). Seven patients declined the intra-articular injection and 4 patients did not complete VAS scoring after the injection.

### Patient-reported outcomes

All patient-reported outcomes, except pain distribution, were collected using the electronic survey software SUNET (Artologic©, Sweden); this was made available to the patients via a link sent by e-mail prior to the clinical examination. The patients rated the perception of their pain, disability and associated problems using the disease-specific questionnaire Copenhagen Hip And Groin Outcome Score (HAGOS), which includes six subscales; pain, symptoms, activities in daily living (ADL), physical function in sport and recreation (Sports/rec), participation in physical activity (PA), and quality of life (QOL). The HAGOS has been proven as a reliable and valid tool in the assessment of LHGP in a young to middle-aged population [28]. The score for each subscale ranges from 0 to 100, where 0 indicates extreme problems and 100 no problems. HAGOS data (mean, 95% CI) from 19 healthy individuals (mean age 27, 42% women) was used as normative values [8].

The patients rated their activity level during adolescence, pre-injury, and current activity level on the Hip Sports Activity Scale (HSAS), which is a valid and reliable questionnaire for assessing activity level in this patient group [29]. To rate their perceived general physical and mental health, the patients completed the Medical Outcomes Study 36-Item Short Form Health Survey (SF-36), which includes eight subscales: physical functioning, physical role functioning, bodily pain, general health perception, vitality, social role functioning, emotional role functioning, and mental health. A combined physical component score and mental component score is also calculated. The score for each subscale ranges from 0 to 100, where 0 indicates extreme problems and 100 no problems [30, 31]. Population sample of SF-36 data (mean, 95% CI) from 5140 individuals (age range 15–44, 52% women) was used as normative values [30]. Nine patients did not complete the HAGOS, HSAS and SF-36 (reason unknown).

To describe pain distribution, each patient completed a pain drawing on a full-body manikin, viewing the front and back separately. The patients were instructed to outline the area of their pain. The pain manikin was then divided into 9 separate areas as previously described [32]:1) lower back, 2) groin, 3) buttock, 4) anterior thigh, 5) posterior thigh, 6) anterior knee, 7) posterior knee, 8) anterior lower leg, and 9) posterior lower leg. The areas were outlined on a transparent plastic sheet. This plastic sheet was then placed on each patient’s pain manikin to identify the painful area(s). The number, and percentage, of patients that had marked each area on the pain manikin were recorded. One patient did not complete the pain manikin drawing (reason unknown).

### Ethics

The Regional Ethical Review Board in Lund approved the study (Dnr 2014/12) and the participants provided written informed consent to participate.

### Statistical analysis

All calculations were performed in SPSS for Windows, version 22.0 (IBM Corp., Armonk, New York, USA). All variables were tested for skewness. The independent t-test was used for between-group analysis of the HAGOS and the SF-36. Comparison of the HAGOS and SF-36 scores of the patients and normative data was performed by calculating the 95% confidence interval for all groups (95 % *CI* =  ± 1.96 × *SE*). For between-group analysis of the HSAS score, the Mann-Whitney U-test was used. Pain distribution was calculated as frequency, percent and 95% confidence interval using the formula $$ 95\% CI=\pm 1.96\times \sqrt{\frac{\begin{array}{c}p\times \Big(1-p\\ {}\Big)\end{array}}{n}} $$. The chi-square test was used to compare between-group differences in gender and pain distribution.

## Results

### Prevalence of hip-related groin pain

Eleven patients had missing data for either radiographs or patient-reported response after block injection, and could therefore not be categorized (Table 2). Seventy patients were categorized as either having hip-related groin pain or non-hip-related groin pain. Thirty-three patients (47%) met all four criteria for hip-related groin pain. Thirty-seven patients (53%) did not meet all four criteria and were categorized as having non-hip-related groin pain.

**Table 2:** The number of patients undergoing the different examinations and the number and percentage of positive and negative results from each examination

	Patients n	Positive result n (%)	Negative result n (%)
Criterion 1			
Affected hip ROM	81	71 (88)	10 (12)
Criterion 2			
Pain during any of the impingement test	81	76 (94)	5 (6)
Criterion 3			
Radiographic data. Alfa angle > 60 (CAM morphology)	75^a^	34 (45)	41 (55)
Radiographic data. LCE angle > 40 (Pincer morphology)	79^b^	19 (24)	60 (76)
Radiographic data. LCE angle < 20 (Dysplasia)	79^b^	1 (1)	78 (99)
Findings on MRI corresponding to hip-related pathology	54^c^	19 (35)	35 (65)
Findings during arthroscopy corresponding to hip-related pathology	19^d^	17 (89)	2 (11)
Criterion 4			
≥ 50% decrease of patient reported pain from intra-articular block injection	70^e^	49 (70)	21 (30)
Criteria 1 + 2 + 3 + 4	70	33 (47)	37 (53)

LCE angle = Lateral center-edge angle

^a^ Missing data (n = 6) due to missing radiographs or missing Lauenstein projection

^b^ Missing data (n = 3) due to missing radiographs

^c^ Missing data (*n* = 27) due to missing clinical relevance for the examination

^d^ Missing data (*n* = 62) due to missing clinical relevance for the examination

^e^ Missing data (*n* = 11) due to either declined injection or failure to complete VAS scoring after the injection

### Patient characteristics

The mean age was 36 years and the mean BMI was 24.82 kg/m^2^. Sixteen percent of the patients reported symptoms in both hips. The group with hip-related groin pain contained more men than the group with non-hip-related groin pain (70% vs 38%, *p* < 0.01). No differences between groups were observed for any other patient characteristics (Table 3).

**Table 3:** Patient characteristics for all patients, patients with hip-related groin pain (HRGP) and patients with non-hip-related groin pain (Non-HRGP), and mean difference between groups with 95% confidence interval (95%CI). Data is expressed as mean and standard deviation (SD) unless otherwise stated

	All patients *n* = 81 Mean (SD)	HRGP *n* = 33 Mean (SD)	Non-HRGP *n* = 37 Mean (SD)	Mean difference (95%CI) (Non-HRGP minus) HRGP
Age (years)	36 (9)	35 (10)	35 (8)	0 (−4; 4)
Gender women (n)/(%)	40/49	10/30	23/62	13/32 (23; 58)
Height (cm)	174.7 (9.6)	176.4 (8.6)	173.3 (14.7)	−3.1 (−7.7; 1.4)
BMI (kg/m^2^)	24.82 (3.92)	25.96 (4.31)	24.21 (3.51)	−1.75 (−3.61; 0.12)
Unilateral symptoms left/right (n)	29/39	16/12	10/23	−6/−11
Bilateral symptoms (n)	13	5	4	−1
Duration of pain (n)/(%)				
• 3–6 months	2/2.5	1/3.0	1/2.7	0/0.3 (0; 1.5)
• 6–12 months	15/18.5	5/15.2	6/16.2	−1/−1 (0; 3.2)
• More than 12 months	16/19.8	7/21.2	8/21.6	−1/−0.4 (0; 1.8)
• Several years	39/48.1	18/54.5	16/43.2	−2/−11.3 (4.4; 18.2)
• Unknown	9/11,1	6/18.2	2/6.7	−4/−11.5 (4.5; 18.5)

### Pain distribution

**Fig. 4:**
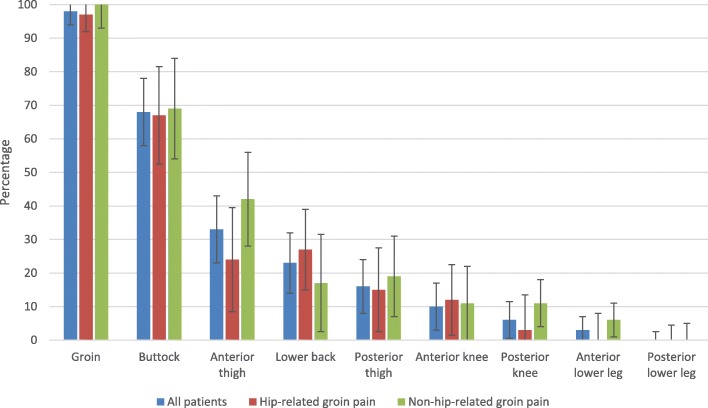
Pain distribution in percent (%) and 95% confidence interval of the different areas for all patients (*n* = 80), patients with hip-related groin pain (*n* = 33), and patients with non-hip-related groin pain (*n* = 36). Missing data, *n* = 1 (did no fill in)

The most common body areas with pain reported by the patients were the groin (98%) and the buttock (68%). Thirty three percent of the patients reported pain in the anterior thigh, and 23% reported low back pain. Pain was less prevalent in the posterior thigh (16%), anterior knee (10%), posterior knee (6%) and anterior lower (3%) leg. No differences were noted between the hip-related and non-hip-related groin pain groups (Fig. 4) (Table 4 Appendix).

### Activity level

**Fig. 5:**
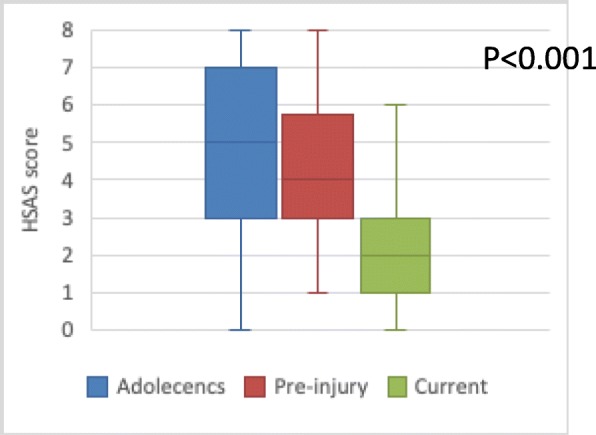
Activity level during adolescence, pre-injury and current activity level in terms of HSAS score with median, first and third quartile and range for all patients (*n* = 72). Missing data, *n* = 9 (did no fill in)

**Fig. 6:**
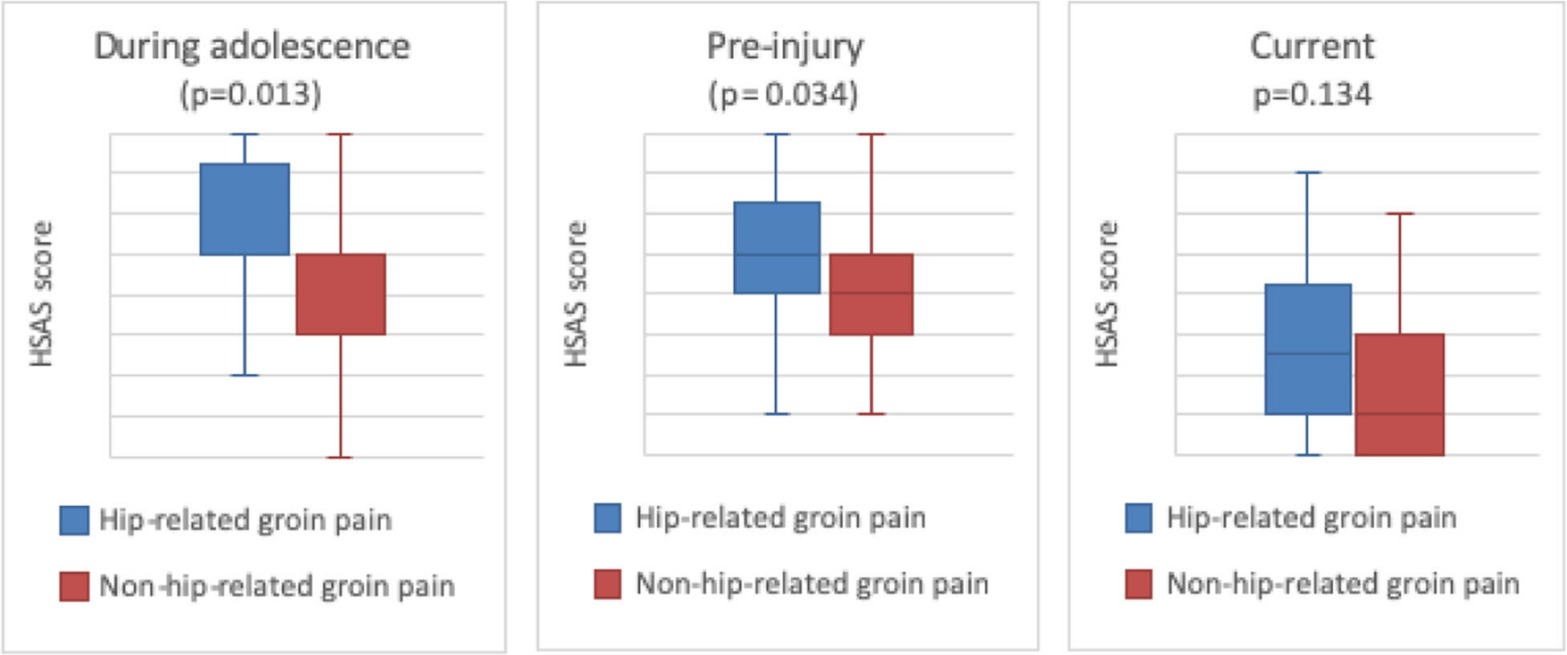
Activity level during adolescence, pre-injury and current activity level in terms of HSAS score with median, first and third quartile and range for patients with hip-related groin pain (*n* = 30), and patients with non-hip-related groin pain (*n* = 32). Missing data, n = 8 (did no fill in)

The pre-injury activity level (median 5, inter-quartal range (IQR) 3–7) and the activity level during adolescence (median 4, IQR 3–5.75) was higher compared to the current activity level (median 2, IQR 1–3) (*p* < 0.001) (Fig. 5) (Table 5 Appendix). The group with hip-related groin pain scored a higher activity level during adolescence (median 5, IQR 5–7.5) as well as a higher pre-injury activity level (median 5, IQR 4–6.25) compared to the group with non-hip-related groin pain (median 5, IQR 3–5/median 4, IQR 3–5) (*p* ≤ 0.034) (Fig. 6). No difference was found in current activity level between the two groups (hip-related groin pain (median 2.5, IQR 1–4.25) vs non-hip-related groin pain (median 1, IQR 0–3))(*p* = 0.134) (Fig. 6) (Table 5 Appendix).

### HAGOS

**Fig. 7:**
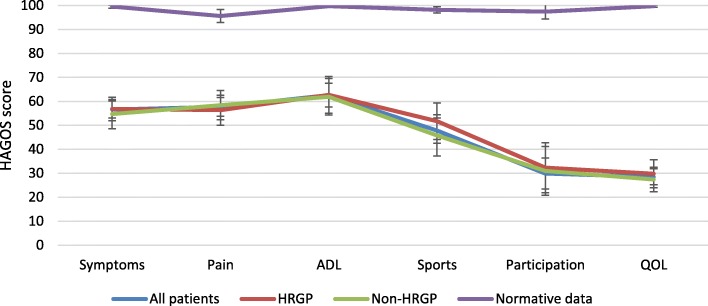
HAGOS score for all patients (*n* = 72), patients with hip-related groin pain (*n* = 30), and patients with non-hip-related groin pain (*n* = 32). Missing data, *n* = 9 (did no fill in). Normative data was extracted from Wörner et al. (*n* = 19) (54)

The worst score on the HAGOS was reported for the subscale Quality of Life (mean 28.5, standard error (SE) 1.7) and the best score was reported for the subscale Activities of Daily Living (mean 62.6, SE 2.5). No differences were found between the groups in any HAGOS subscale, however, compared to normative data the patients had worse score in all subscales (Fig. 7) (Table 6 Appendix).

### Sf-36

**Fig. 8:**
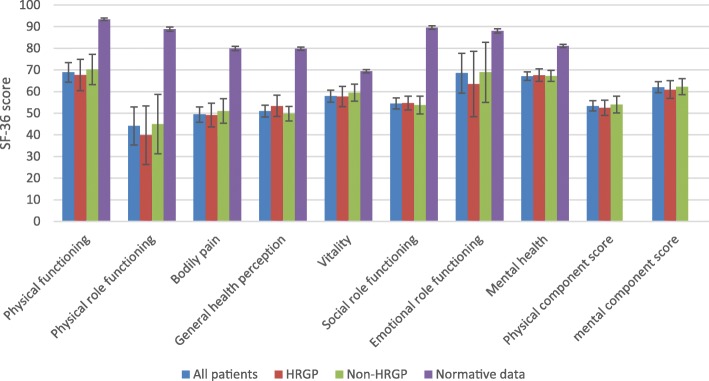
SF-36 mean score and 95% confidence interval for all patients (*n* = 72), patients with hip-related groin pain (n = 30), and patients with non-hip-related groin pain (*n* = 32). Missing data, *n* = 9 (did no fill in). Normative data was extracted from Sullivan et al. (*n* = 5140) [30]

For SF-36, patients reported the worst score for the subscale physical role functioning (mean 44.1, SE 4.5) and the best score for the subscale Physical functioning (mean 68.9, SE 2.3). No differences were observed between the groups in any SF-36 subscale, however, compared to normative data the patients reported worse score in all subscales (Fig. 8) ( Table 7 Appendix).

## Discussion

In this exploratory, cross-sectional, study, 47% of the patients with LHGP referred to tertiary care, of which 70% were men, were categorized as having hip-related groin pain. All of the patient groups had a lower current activity level compared to pre-injury. Patients with hip-related groin pain had a higher activity level during adolescence, and a higher activity level pre-injury, compared to those with non-hip-related groin pain. The patients had worse patient-reported outcomes in terms of generic health and disease-specific symptoms and function compared to normative data, but no differences were noted between the patients with hip-related and those with non-hip-related groin pain. The most common pain localizations were the groin and buttock, followed by the anterior thigh and lower back, with no differences between the groups.

The prevalence of approximately 50% of hip-related groin pain in our cohort is in line with a study by Larson et al. [13]. They reported a prevalence of hip-related groin pain of 57% in a cohort of 499 consecutive patients (54% women, mean age 38 years) with LHGP referred to tertiary care [13].

In that study [13], the authors used the same diagnostic criteria for identifying hip-related groin pain as those used in our present study. Although further studies are needed to confirm these findings, the results from our study, and those of Larsen et al. [13], suggest that approximately 50% of patients may not need to be referred to orthopedic surgeons, due to lack of indication for surgery.

The Warwick agreement provides the current best criteria for identifying hip-related groin pain and suggests that a combination of clinical symptoms, signs, and radiological findings should be used [17]. However, Peters et al. [33] argued in a recent scoping review of surgical criteria for FAI syndrome that only 56% of the systematically included studies utilized the criteria stated in the Warwick agreement, and that the diagnosis often was based solely on radiological findings. Using only radiological findings as a diagnostic criterion could lead to an over-estimation of hip-related groin pain. This is because hip CAM, pincer morphology and MRI findings of hip pathology, such as labral tears and chondral lesions, can be present in the asymptomatic population [23, 34, 35] and are common in asymptomatic athletes [36, 37]. Our findings that only about half of the patients referred to tertiary care were potential candidates for surgery could indicate that the diagnostic criteria according to current best evidence have not been used in primary care to refer the patients to tertiary care. By applying and implementing the diagnostic criteria in primary care, treatment management can be optimized so that the appropriate patients are referred to tertiary care for consideration of arthroscopic surgery. However, only a subgroup of patients with hip-related groin pain might benefit from a combination of hip surgery and exercise-based therapy [19]. All patients should primarily be managed with education, modification of activity level, and exercise-based therapy [17, 38] while waiting for assessment for hip surgery.

In the present study, more men than women were categorized as having hip-related groin pain. The prevalence of FAI morphology, especially CAM morphology, has been reported to be higher in men than women [35]. One potential reason for this is that CAM morphology is a consequence of slipped capital femoral epiphysis during adolescence, which is predominantly a male condition [39, 40]. It is also believed that participation in high-impact sports such as soccer, basketball, and ice hockey during adolescence is a risk factor for development of FAI syndrome, where the high impact and training intensity might lead to development of CAM morphology during skeletal maturation [41–44]. Our results of a high activity level during adolescence in the group with hip-related groin pain could support that theory. However, prospective longitudinal studies are needed to evaluate whether a high activity level in adolescence is a risk factor for development of future hip-related groin pain. Moreover, the higher pre-injury activity level in patients with hip-related groin pain could indicate that these patients have higher physical demands, and that both previous and desired activity level should be considered in the exercise-based therapy for these patients. Although we showed statistical differences between the groups in activity level, the clinical relevance can be questioned since the HSAS score does not provide information about actual hip load or intensity, frequency or duration of the activities [29].

The patients in our study reported worse outcomes with significant impairments in both the HAGOS and the SF-36 compared to healthy people of the same age. The patients reported the worst score on the HAGOS subscale quality of life. Reduced quality of life has also been shown in patients 12–24 months post hip arthroscopy [45]. Perceived low quality of life can be due to pain, low physical function and/or being unable to maintain a desired physical activity level [46], but may also be due to psychosocial challenges. Nisar et al. [47] found a significantly higher level of depression and anxiety in a cohort of 49 patients referred to tertiary care for hip pain, compared with an asymptomatic population. Although the level of anxiety and prevalence of depressive symptoms is unknown in our cohort, these could be important factors influencing the patient’s perceived quality of life and could thus be a subject for further study. The poor generic health and disease-specific outcome scores reported by both patients with hip-related groin pain and those with non-hip-related groin pain, indicate that early optimal treatment options for all patients are needed to improve general health and improve hip-related symptoms and function. Both patient-reported outcomes and objective tests of physical function are important to obtain a complete picture of the patient’s function [48]. Therefore, to further optimize treatment for patients with LHGP, especially exercise-based therapy, more information is required on possible limitations in their physical function.

The patient reported pain distribution in our cohort, with predominantly proximal pain (groin and buttock) and pain to the anterior thigh and knee, is similar to the distribution of pain described by patients with hip osteoarthritis [32]. The pain reported to the anterior thigh can be explained by the sensory distribution of the femoral and obturator nerves, which also innervate the hip joint [49, 50]. The similarity in pain localization and distribution between the two groups means that reported pain cannot be used to distinguish whether a patient has hip-related or non-hip-related groin pain. Although patients with low back/spine pathology were excluded from the study, almost one in four patients (23%) reported low back pain. Although hip ROM was not measured in degrees with a technical device, 94% of the patients in the present study had affected hip ROM in terms of decreased and/or end-range pain. One reason for the high prevalence of low back pain in our cohort could be a consequence of limited hip ROM. One reason for the high prevalence of low back pain in our cohort could be a consequence of limited hip ROM. Limited hip ROM has been found in cohorts of patients with low back pain [51–53], where the authors hypothesized that limited hip range of motion might lead to compensatory movement with premature and greater lumbopelvic movement, thereby increasing the load on the spine. The high prevalence of low back pain in patients with LHGP should be considered in the treatment of these patients.

A strength of our study is that we consecutively included patients referred to the Orthopedic Department of a University Hospital serving a regional area (Skåne county) with a population of approximately 1.3 million residents, and the absence of any private clinics offering arthroscopic hip surgery. Therefore, the patients are likely to be representative for the clinical setting in tertiary care. However, 11 patients were not categorized due to missing data, which might have influenced the results regarding the prevalence and the comparison between the two patient groups. A limitation is that the prevalence of hip-related groin pain is not generalizable to primary care. The majority of the patients included in this study were referred to the orthopedic surgeon for assessment for hip surgery. Therefore, the probability of patients having hip-related groin pain in this group is expected to be higher compared to the population with LHGP in primary care. Another strength is that we used the current best evidence to categorize the patients as having hip-related or non-hip-related groin pain [13, 17]. However, the reliability and construct validity of these combined criteria to identify hip-related groin pain need to be determined and should, thus, be a subject for further study.

## Conclusions

Only half of the patients referred to tertiary care for long standing hip and groin pain, who were predominantly men with a high activity level, had hip-related groin pain. Self-reported pain localization and distribution did not differ between patients with hip-related groin pain and those with non-hip-related groin pain, and both patient groups worse perceived general health, and hip-related symptoms and function compared with healthy people of the same age. To further optimize treatment management for patients with LHGP, diagnostic criteria should be implemented in primary care, so that appropriate patients are referred to tertiary care. Also early optimal treatment options, especially exercise-based treatment, for all patients are needed to improve general health and improve hip-related symptoms and function.

## Data Availability

The datasets used and analyzed during the current study are available from the corresponding author on reasonable request.

## Appendix

**Table 4:** Pain distribution in frequency (n), percent (%) and 95% CI of the different body areas

	All patients *n* = 80*		HRGP *n* = 33		Non-HRGP *n* = 36	
	n (%)	95% CI	n (%)	95% CI	n (%)	95% CI
Groin	78 (98)	85–99	32 (97)	91–99	36 (100)	90–100
Buttock	54 (68)	50–80	22 (67)	57–77	25 (69)	53–82
Anterior thigh	26 (33)	13–41	8 (24)	23–43	15 (42)	27–58
Lower back	18 (23)	15–44	9 (27)	15–33	6 (17)	8–32
Posterior thigh	13 (16)	7–31	5 (15)	10–26	7 (19)	10–35
Anterior knee	8 (10)	5–27	4 (12)	5–19	4 (11)	4–25
Posterior knee	5 (6)	1–15	1 (3)	3–14	4 (11)	4–25
Anterior lower leg	2 (3)	0–10	0 (0)	1–9	2 (6)	2–18
Posterior lower leg	0 (0)	0–10	0 (0)	0–5	0 (0)	0–9

* One patient did not complete the pain manikin

**Table 5:** HSAS score for all patients, patients with hip-related groin pain (HRGP). and patients with non-hip-related groin pain (Non-HRGP). Data is expressed as median and interquartile range (IQR). Nine patients did not complete the HSAS

	All patients Median (IQR)	HRGP Median (IQR)	Non-HRGP Median (IQR)
*HSAS*	n = 72	n = 30	n = 32
*HSAS adolescent (IQR)*	5 (3–7)	5 (5–7,5)	5 (3–5)
*HSAS pre injury median (IQR)*	4 (3–5.75)	5 (4–6.25)	4 (3–5)
*HSAS current median (IQR)*	2 (1–3)	2.5 (1–4.25)	1 (0–3)

HSAS = Hip Sports Activity Scale

**Table 6:** HAGOS score for all patients, patients with hip-related groin pain (HRGP). and patients with non-hip-related groin pain (Non-HRGP). Mean difference between groups and 95% confidence interval (95%CI). Data is expressed as mean and standard error (SE). Nine patients did not complete the HAGOS

	All patients Mean (SE)	HRGP Mean (SE)	Non-HRGP Mean (SE)	Mean difference (95%CI) Non-HRGP - HRGP
*HAGOS scores*	n = 72	n = 30	n = 32	
*Symptoms*	56.6 (1.8)	54.7 (2.5)	56.8 (3.1)	2.1 (−5.9; 10.0)
*Pain*	57.7 (2.0)	58.4 (3.2)	56.3 (3.1)	−2.1 (−10.9; 6.7)
*ADL*	62.6 (2.5)	61.9 (3.9)	62.7 (3.9)	0.8 (−10.2; 11.8)
*Sport/Rec*	47.8 (2.7)	45.8 (3.9)	51.7 (4.4)	5.9 (−5.9; 17.8)
*PA*	29.9 (3.3)	31.0 (5.3)	32.3 (5.2)	1.2 (−13.6; 16.0)
*QOL*	28.5 (1.7)	27.4 (3.0)	29.8 (2.6)	2.4 (−5.5; 10.3)

HAGOS = Copenhagen Hip And Groin Outcome Score, ADL = Activities of Daily Living, PA = Physical Activities, QOL = Quality Of Life

**Table 7:** SF-36 score for all patients, patients with with hip-related groin pain (HRGP). and patients with non-hip-related groin pain (Non-HRGP). Mean difference between groups and 95% confidence interval (95%CI). Data is expressed as mean and standard error (SE). Nine patients did not complete the SF-36

	All patients Mean (SE)	HRGP Mean (SE)	Non-HRGP Mean (SE)	Mean difference (95%CI) Non-HRGP - HRGP
*SF-36*	n = 72	n = 30	n = 32	
*Physical functioning*	68.9 (2.3)	70.2 (3.7)	67.7 (3.6)	−2.5 (−12.9; 7.9)
*Physical role functioning*	44.1 (4.5)	45.0 (6.9)	39.8 (7.0)	−5.2 (−24.9; 14.6)
*Bodily pain*	49.4 (1.8)	51.0 (2.8)	49.1 (2.9)	−1.9 (−10.0; 6.1)
*General health perception*	51.0 (1.4)	49.8 (2.5)	53.4 (1.7)	3.6 (−2.3; 9.5)
*Vitality*	57.9 (1.4)	59.5 (2.4)	57.7 (2.0)	−1.8 (−8.1; 4.4)
*Social role functioning*	54.5 (1.3)	53.8 (1.6)	54.7 (2.1)	0.9 (−4.4; 6.2)
*Emotional role functioning*	68.5 (4.7)	68.9 (7.7)	63.5 (7.1)	−5.3 (−26.2; 15.5)
Mental health	67.1 (1.0)	67.2 (1.5)	67.6 (1.3)	0.4 (−3.5; 4.4)
Physical component score	53.4 (1.2)	54.0 (1.8)	52.5 (2.0)	−1.5 (−6.9; 3.9)
Mental component score	62.0 (1.3)	62.3 (2.1)	60.9 (1.9)	−1.5 (−7.1; 4.2)

SF-36 = Medical Outcomes Study 36-Item Short Form Health Survey

## Abbreviations

95%CI: 95% confidence interval
ADL: Activities in daily living
AIMT: Anterior Impingement Test
DEXRIT: Dynamic External Rotatory Impingement Test
DIRIT: Dynamic Internal Rotatory Impingement Test
FABER: Flexion/Abduction/External Rotation
FADIR: Flexion/Adduction/Internal Rotation
FAI: Femoroacetabular impingement
HAGOS: Copenhagen Hip and Groin Outcome Score
HRGP: Hip-related groin pain
HSAS: Hip Sports Activity Scale
IQR: Inter-quartal range
LCE: Lateral center-edge
LHGP: Long-standing hip and groin pain
Non-HRGP: Non-hip-related groin pain
OA: Osteoarthritis
PA: Physical activity
PRIMT: Posterior Rim Impingement Test
QOL: Quality of life
ROM: Range of motion
SD: Standard deviation
SF-36: Medical Outcomes Study 36-Item Short Form Health Survey
Sports/rec: Sport and recreation
VAS: Visual analog scale
